# The Landscape of Pediatric Acute Care Nephrology Programs: A National Survey from the American Society of Pediatric Nephrology

**DOI:** 10.34067/KID.0000000593

**Published:** 2024-09-26

**Authors:** Keri Drake, Shina Menon, Kara Short, Katie Plomaritas, Brendan Crawford, Kyle Merrill, Alyssa Riley, Weiwen Vivian Shih, David Askenazi, David Selewski

**Affiliations:** 1 UT Southwestern Medical Center, Dallas, Texas; 2 Division of Nephrology, Department of Pediatrics, Stanford University, Palo Alto, California; 3 Children's of Alabama, University of Alabama at Birmingham, Birmingham, Alabama; 4 C.S Mott Children's Hospital, Ann Arbor, Michigan; 5 Arkansas Children's Hospital, Little Rock, Arkansas; 6 Division of Pediatric Nephrology, Department of Pediatrics, University of Iowa, Iowa City, Iowa; 7 Nationwide Children's Hospital – Toledo, Toledo, Ohio; 8 University of Colorado School of Medicine, Denver, Colorado; 9 Department of Pediatrics, Medical University of South Carolina, Charleston, South Carolina

**Keywords:** children

## Introduction

The past 20 years of research have defined the epidemiological burden and deleterious effect of AKI and fluid overload, clearly showing their independent contribution to increased morbidity and mortality in critically ill neonates and children.^1–3^ Renal replacement therapy (RRT), including peritoneal dialysis, intermittent hemodialysis, and continuous RRT (CRRT), is often required for the most severe cases of AKI, with increasing use of Carpediem (Mozarc Medical, Minneapolis) and Aquadex (Nuwellis Inc, Minneapolis) in the neonatal population.^4–6^ These advances in RRT modalities, combined with the challenges of providing multidisciplinary care for medically complex patients, have resulted in a rapidly growing need for critical care nephrology expertise and support across pediatric, neonatal, and cardiac intensive care units.

Understanding the structure of hospital teams and programs to support pediatric-specific aspects of delivering high-quality RRT was recently identified as a critical knowledge gap by the 26th Acute Disease Quality Initiative Consensus Conference.^7^ As institutions have increasingly developed and implemented such dedicated programs, the number of patients, complexity of therapies, and number of available devices have expanded, further heightening the need for structured leadership, education, standardized practices, and quality improvement (QI) programs. While previous reports have explored such practices in adults and resource-limited settings, there remains a paucity of data on the current programmatic support for pediatric and neonatal CRRT. To address this gap, the Acute Care Nephrology (ACN) Interest Group within the American Society of Pediatric Nephrology surveyed high-volume, pediatric institutions across the United States to define current practices developed by individual hospitals/programs, including available infrastructure, resources, and nursing models in supporting the delivery of pediatric acute RRT.

## Methods

The survey was developed by a multidisciplinary team of physicians, advanced practice providers, and nurses and included questions evaluating programmatic structure, support, therapies provided, volume of procedures, QI, and educational practices (Supplemental Material). The questions were reviewed and refined through an iterative process. Here, we defined an ACN program as a “dedicated program with leadership/resources, directed at overseeing the delivery of acute RRT, and focused on critical care nephrology and/or intensive care unit patients.” The final survey was approved by the Institutional Review Board at UT Southwestern Medical Center (STU-2023-0102) with waiver of consent. It was electronically distributed to the division directors of the top 50 pediatric nephrology centers in the US News and World Report Best Children's Hospital 2023. These centers were selected with the goal of reaching high-volume pediatric nephrology centers. The survey could be answered by the division director or their designee. Study data were collected and managed using Research Electronic Data Capture tools hosted at UT Southwestern Medical Center. Data are presented as median (interquartile range) and number (%). Center volumes were reported by total CRRT days in the previous year. Basic statistical analyses comparing centers by volume were performed using R 4.3.1 (R Foundation for Statistical Computing, Vienna, Austria).

## Results

A total of 47 of the 50 eligible centers (94%) completed the survey. Of those, 37 (79%) respondents reported having a dedicated ACN program (Table 1). The ACN program is jointly managed within the chronic dialysis program in 25/37 (68%). Notably, 32 (86%) of the ACN programs have a medical director with a median (interquartile range) full-time equivalent (FTE) of 10% (5, 20%) dedicated to this role, while 6 (19%) programs have no protected effort for the medical director. Twenty-one (57%) of ACN programs have an ACN nursing director. Other available personnel in such programs include nurse educator in 25 (68%), program administrator in 8 (22%), advanced practice provider in 6 (16%), and QI specialist in 10 (27%) programs. Most programs (94%) provide CRRT to neonates aged younger than 30 days, with over half of them using either Carpediem or modified continuous venovenous hemofiltration with Aquadex. Sixty percent of centers have established neonatal CRRT programs, and 28% are either in the process of starting one or plan to start in the next 12 months. From a QI perspective, 19 (40%) of hospitals have ACN-focused QI programs, whereas 16 (36%) do not track any data. Barriers identified for implementing QI programs include lack of protected/dedicated time (85.7%), lack of resources/infrastructure (78.6%), lack of support from leadership (43%), lack of standardized practices (18%), lack of expertise/training (14.3%), lack of interest from providers (10.7%), and low patient volume (10.7%).

**Table 1:** Characteristics of surveyed hospitals/pediatric centers

ACN Programs (*N*=47)	No. (%)
Dedicated ACN program	37 (79)
**Patient volume: CRRT days over the last year (*N*=47), d**	
<250 CRRT	9 (19)
250–499 CRRT	14 (30)
500–749 CRRT	10 (21)
750–999 CRRT	4 (9)
>1000 CRRT	8 (17)
Unknown/do not track	2 (4)
**Location of CRRT (*N*=47)**	
CRRT in PICU	47 (100)
CRRT in NICU	32 (68)
CRRT in CICU	43 (91)
**Neonatal device availability (*N*=47)**	
CRRT performed in neonates <30 d of age	44 (94)
Carpediem	19 (40)
Aquadaex for ultrafiltration	32 (15)
Aquadaex with modified CVVH	14 (30)
**Extracorporeal therapies provided by nephrology (*N*=47)**	
MARS	4 (8)
Single pass albumin dialysis	4 (8)
Plasmapheresis	17 (36)
LDL apheresis	6 (13)
Photopheresis	5 (10)
WBC depletion	4 (8)
RBC apheresis	7 (15)
**Program details: structure of ACN programs (*N*=37)**	
ACN program is part of the chronic dialysis program	25 (68)
Have a dedicated ACN QI program	19 (51)
Apheresis provided by ACN and/or pediatric nephrology	17 (46)
Have an established neonatal CRRT program for >1 yr	28 (76)
Have CRRT competencies for providers	12 (32)
Have CRRT competencies for nurses administering CRRT	30 (81)
**Program details: leadership and ancillary support of ACN programs (*N*=37)**	
Medical director	32 (86)
Nursing director	21 (57)
Nurse educator	25 (68)
Dedicated app	6 (16)
QI leader	10 (27)
ACN program administrator	8 (22)
Pharmacist dedicated to CRRT	20 (54)
Dietitian with specific CRRT experience/training	17 (46)
**Program details: leadership effort (*N*=37), median (IQR)**	
% FTE for current ACN medical director	10 (5–20)
% FTE needed for ACN medical director	20 (15–25)
% FTE for current ACN nursing director	30 (20–50)
% FTE needed for ACN nursing director	30 (20–50)

ACN, acute care nephrology; CICU, cardiovascular intensive care unit; CRRT, continuous renal replacement therapy; CVVH, continuous venovenous hemofiltration; FTE, full-time equivalent; IQR, interquartile range; MARS, molecular adsorbent recirculating system; NICU, neonatal intensive care unit; PICU, pediatric intensive care unit; QI, quality improvement; RBC, red blood cell; WBC, white blood cell.

Given the variability in program sizes surveyed, responses were additionally stratified by patient volume, defined as estimated patient days of CRRT performed in the last calendar year (Table 2). There were no significant differences in the proportion of centers with dedicated ACN programs based on patient volume (*P* value 0.48). Low-volume programs (*i.e*., <250 patient days/year) were less likely to perform CRRT in the neonatal intensive care unit (*P* value = 0.08). Fewer low-volume programs had a medical or nursing director or QI leader compared with programs with higher patient volume. Irrespective of patient volume, survey respondents consistently endorsed an increased need for medical and nursing director FTE.

**Table 2:** Acute care nephrology programs/practices at centers by patient volume

Programmatic Size (*i.e*., Patient Days of CRRT Performed in the Last Calendar Year)	<250	250–499	500–749	750–999	≥1000
No. of centers by patient volume, *N* (%)	9 (19)	14 (30)	10 (21)	4 (9)	8 (17)
Have a dedicated ACN program, *N* (%)	6 (66)	9 (64)	10 (100)	4 (100)	6 (75)
ACN program is part of chronic dialysis, *N* (%)	5 (55)	9 (64)	5 (50)	1 (25)	3 (38)
Have a medical director, *N* (%)	3 (33)	10 (71)	7 (70)	4 (100)	7 (88)
Current FTE for medical director, median (IQR)	20% (12.5–23)	6% (0–14)	10% (8–10)	12.5% (8–17.5)	15% (10–20)
Needed FTE for medical director, median (IQR)	21% (10–25)	20% (15–25)	22.5% (16–32)	17.5% (14–21)	20% (18–26)
Have a nursing director, *N* (%)	2 (22)	5 (35)	4 (40)	4 (100)	5 (63)
Current FTE for nursing director, median (IQR)	10% (5–15)	50% (5–100)	45% (36–60)	29% (25–48)	40% (20–40)
Needed FTE for nursing director, MEDIAN (IQR)	20% (20–25)	50% (20–50)	28% (25–50)	50% (44–63)	50% (24–62)
Have dedicated app(s), *N* (%)	0	2 (14)	0	1 (25)	3 (38)
Have a nurse educator, *N* (%)	3 (33)	6 (42)	9 (90)	3 (75)	3 (38)
Have a program administrator, *N* (%)	0	3 (21)	1 (10)	1 (25)	2 (25)
Have a dedicated pharmacist, *N* (%)	3 (33)	3 (21)	2 (20)	2 (50)	6 (75)
Have a QI program, *N* (%)	2 (22)	4 (29)	5 (50)	3 (75)	5 (63)
Have a QI leader, *N* (%)	0	4 (29)	3 (30)	1 (25)	2 (25)
Have a dedicated ACN nurse(s), *N* (%)	1 (11)	1 (7)	1 (10)	3 (75)	5 (63)
Perform CRRT in the NICU, *N* (%)	2 (22)	10 (71)	9 (90)	3 (75)	6 (75)
Perform CRRT in the CICU, *N* (%)	6 (66)	14 (100)	10 (100)	4 (100)	8 (100)
Perform CRRT through Carpediem, *N* (%)	5 (55)	5 (35)	4 (40)	2 (50)	5 (63)
Perform ultrafiltration through Aquadex, *N* (%)	3 (33)	5 (35)	1 (10)	3 (75)	4 (50)
Perform modified CVVH through Aquadex, *N* (%)	0	4 (29)	1 (10)	3 (75)	4 (50)
Perform mars, *N* (%)	1 (11)	1 (7)	0	1 (25)	1 (13)
Plasmapheresis managed by nephrology, *N* (%)	0	5 (35)	3 (30)	1 (25)	7 (88)
LDL apheresis managed by nephrology, *N* (%)	1 (11)	1 (7)	1 (10)	1 (25)	2 (25)

ACN, acute care nephrology; CICU, cardiovascular intensive care unit; CRRT, continuous renal replacement therapy; CVVH, continuous venovenous hemofiltration; FTE, full-time equivalent; IQR, interquartile range; NICU, neonatal intensive care unit; QI, quality improvement.

## Discussion

The management of patients with AKI requires early identification and timely interventions, relying on collaboration between nephrologists and intensivists, a multidisciplinary team, and hospital support to optimize the delivery of care for these complex, acutely ill patients.^8^ While the importance of such multidisciplinary coordination of care has been long recognized,^9–11^ the challenges of reliably providing safe, effective, and timely therapies have only grown with the increasing complexity of pediatric patients and availability of newer, pediatric-focused devices (Carpediem and Aquadex). Here, we present the first detailed report of ACN programs developed by US institutions to address these growing needs and work toward optimizing the delivery of care in pediatric RRT practices.

The development of standardized recommendations in the delivery of acute pediatric dialysis^12^ has lagged behind other care models (such as chronic dialysis and transplant). Given this lack of standardization, this survey was designed to broadly capture how individual institutions have thus far developed their own unique organizational structure and systematic practices. Our findings demonstrate important variations in the current modalities of RRT offered, programmatic structure, effort and/or ancillary support, as well as QI capacity. We not only describe the current programmatic structure at large, reputable pediatric nephrology centers across the US, but also identify important opportunities and critical gaps for improved programmatic development.

Accordingly, this survey identified several concerning findings. While most of the ACN programs have a medical director, 45% of them have no or <10% FTE protected time for this work. Most programs reported a need for increased medical and nursing director FTEs. Interestingly, we noted a discrepancy in the number of centers performing CRRT in neonates compared with those performing CRRT in the neonatal intensive care unit. This is likely because of some centers transferring neonates to other units (*i.e*., cardiovascular intensive care unit, pediatric intensive care unit) when CRRT is required, as has been previously reported.^13^ Typically this is due to lower volumes of neonatal CRRT in the centers, lack of familiarity, low comfort with these procedures by neonatologists, and challenges with maintaining nursing competency. This is likely to change with increasing use of neonatal-specific dialysis devices.

Importantly, only 40% of responding centers have a dedicated QI program, while 36% of centers do not track any data related to quality assessment/improvement in their pediatric CRRT patients. Given that such data metrics provide the foundation for the delivery of better patient care, bolster quality assessment and practice improvement, and give opportunities for participation in multicenter research, this remains a critically important area of opportunity. Furthermore, the worsening pediatric nephrology workforce crisis,^14^ coupled with increasing demands for expertise, oversight, and leadership in pediatric/neonatal RRT, may hinder the ability of programmatic support of institutions in delivering high-quality RRT, performing quality assessments, and participating in multicenter trials to advance the field.

While the current state of ACN programs has created exciting opportunities for standardizing care and making progress toward developing evidence-based guidelines and best practice recommendations, such clinically important initiatives can only be achieved with dedicated resources and support, which must be at the forefront of priorities for individual hospitals and institutions, as well as national pediatric health care agendas and policies.

## Supplementary Material

SUPPLEMENTARY MATERIAL

## Data Availability

Partial restrictions to the data and/or materials apply. Data from may be shared after approval with IRB and with appropriate data use agreement.

## Supplemental Material

Pediatric Acute Renal Replacement Survey
