# Supplementary material for: The Landscape of Pediatric Acute Care Nephrology Programs: A National Survey from the American Society of Pediatric Nephrology
Source: Kidney360. 2024 Sep 26;5(11):1713–7. doi: 10.34067/KID.0000000593 (PMC12282623; doi:10.34067/KID.0000000593)
Supplement: SUPPLEMENTARY MATERIAL [file kidney360-5-1713-s001.pdf]

ASN American Society of Nephrology JASN Journal of the American Society of Nephrology KIDNEY360 Accessing Our World From Every Angle CJASN Clinical Journal of the American Society of Nephrology

ASN Journal Disclosure Form

As per ASN journal policy, I have disclosed any financial relationships or commitments I have held in the past 36 months as included below. I have listed my Current Employer below to indicate there is a relationship requiring disclosure. If no relationship exists, my Current Employer is not listed.

D. Askenazi reports the following:
Employer: University of Alabama at Birmingham; Consultancy: Nuwellis, Abbott, Seastar; Ownership Interest: Zorro-FLow Inc.; Research Funding: Nuwellis, Seastar, Bioporto; Patents or Royalties: Pending patents on a female urine collection device, Improvements to CRRT systems, Fluid Aviator.; and Advisory or Leadership Role: Chief Scientific Officer - Zorro-Flow.

I understand that the information above will be published within the journal article, if accepted, and that failure to comply and/or to accurately and completely report the potential financial conflicts of interest could lead to the following: 1) Prior to publication, article rejection, or 2) Post-publication, sanctions ranging from, but not limited to, issuing a correction, reporting the inaccurate information to the authors' institution, banning authors from submitting work to ASN journals for varying lengths of time, and/or retraction of the published work.

Name: David J. Askenazi
Manuscript ID: K360-2024-000578R1
Manuscript Title: The Landscape of Pediatric Acute Care Nephrology Programs: A National Survey from the American Society of Pediatric Nephrology"
Date of Completion: August 29, 2024
Disclosure Updated Date: August 29, 2024

ASN American Society of Nephrology | JASN Journal of the American Society of Nephrology | KIDNEY360 Accessing Our World From Every Angle | CJASN Clinical Journal of the American Society of Nephrology

ASN Journal Disclosure Form

As per ASN journal policy, I have disclosed any financial relationships or commitments I have held in the past 36 months as included below. I have listed my Current Employer below to indicate there is a relationship requiring disclosure. If no relationship exists, my Current Employer is not listed.

B. Crawford reports the following:
Employer: University Arkansas Medical Sciences

I understand that the information above will be published within the journal article, if accepted, and that failure to comply and/or to accurately and completely report the potential financial conflicts of interest could lead to the following: 1) Prior to publication, article rejection, or 2) Post-publication, sanctions ranging from, but not limited to, issuing a correction, reporting the inaccurate information to the authors' institution, banning authors from submitting work to ASN journals for varying lengths of time, and/or retraction of the published work.

Name: Brendan Crawford
Manuscript ID: K360-2024-000578R1
Manuscript Title: The Landscape of Pediatric Acute Care Nephrology Programs: A National Survey from the American Society of Pediatric Nephrology
Date of Completion: September 7, 2024
Disclosure Updated Date: August 28, 2024

ASN American Society of Nephrology · JASN Journal of the American Society of Nephrology · KIDNEY360 Accessing Our World From Every Angle · CJASN Clinical Journal of the American Society of Nephrology

ASN Journal Disclosure Form

As per ASN journal policy, I have disclosed any financial relationships or commitments I have held in the past 36 months as included below. I have listed my Current Employer below to indicate there is a relationship requiring disclosure. If no relationship exists, my Current Employer is not listed.

K. Drake reports the following:
Employer: UT Southwestern Medical Center

I understand that the information above will be published within the journal article, if accepted, and that failure to comply and/or to accurately and completely report the potential financial conflicts of interest could lead to the following: 1) Prior to publication, article rejection, or 2) Post-publication, sanctions ranging from, but not limited to, issuing a correction, reporting the inaccurate information to the authors' institution, banning authors from submitting work to ASN journals for varying lengths of time, and/or retraction of the published work.

Name: Keri A. Drake
Manuscript ID: K360-2024-000578R1
Manuscript Title: The Landscape of Pediatric Acute Care Nephrology Programs: A National Survey from the American Society of Pediatric Nephrology
Date of Completion: August 28, 2024
Disclosure Updated Date: August 28, 2024

ASN American Society of Nephrology | JASN Journal of the American Society of Nephrology | KIDNEY360 Accessing Our World From Every Angle | CJASN Clinical Journal of the American Society of Nephrology

ASN Journal Disclosure Form

As per ASN journal policy, I have disclosed any financial relationships or commitments I have held in the past 36 months as included below. I have listed my Current Employer below to indicate there is a relationship requiring disclosure. If no relationship exists, my Current Employer is not listed.

S. Menon reports the following:
Employer: Stanford University; IOV Labs (Spouse); Consultancy: Nuwellis Inc (CHF Solutions);; Ownership Interest: Zorroflow Inc; Research Funding: Bioporto; Gerber Foundation; Honoraria: Medtronic Inc; and Advisory or Leadership Role: Board Member, Neonatal Kidney Collaborative.

I understand that the information above will be published within the journal article, if accepted, and that failure to comply and/or to accurately and completely report the potential financial conflicts of interest could lead to the following: 1) Prior to publication, article rejection, or 2) Post-publication, sanctions ranging from, but not limited to, issuing a correction, reporting the inaccurate information to the authors' institution, banning authors from submitting work to ASN journals for varying lengths of time, and/or retraction of the published work.

Name: Shina Menon
Manuscript ID: K360-2024-000578R1
Manuscript Title: The Landscape of Pediatric Acute Care Nephrology Programs: A National Survey from the American Society of Pediatric Nephrology
Date of Completion: August 30, 2024
Disclosure Updated Date: May 16, 2024

ASN American Society of Nephrology | JASN Journal of the American Society of Nephrology | KIDNEY360 Accessing Our World From Every Angle | CJASN Clinical Journal of the American Society of Nephrology

ASN Journal Disclosure Form

As per ASN journal policy, I have disclosed any financial relationships or commitments I have held in the past 36 months as included below. I have listed my Current Employer below to indicate there is a relationship requiring disclosure. If no relationship exists, my Current Employer is not listed.

K. Merrill reports the following:
Employer: University of Iowa Healthcare; and Consultancy: BioPorto Diagnostics.

I understand that the information above will be published within the journal article, if accepted, and that failure to comply and/or to accurately and completely report the potential financial conflicts of interest could lead to the following: 1) Prior to publication, article rejection, or 2) Post-publication, sanctions ranging from, but not limited to, issuing a correction, reporting the inaccurate information to the authors' institution, banning authors from submitting work to ASN journals for varying lengths of time, and/or retraction of the published work.

Name: Kyle Merrill
Manuscript ID: K360-2024-000578R1
Manuscript Title: The Landscape of Pediatric Acute Care Nephrology Programs: A National Survey from the American Society of Pediatric Nephrology
Date of Completion: August 28, 2024
Disclosure Updated Date: August 28, 2024

ASN
American Society of Nephrology
JASN
Journal of the American Society of Nephrology
KIDNEY360
Accessing Our World From Every Angle
CJASN
Clinical Journal of the American Society of Nephrology

ASN Journal Disclosure Form

As per ASN journal policy, I have disclosed any financial relationships or commitments I have held in the past 36 months as included below. I have listed my Current Employer below to indicate there is a relationship requiring disclosure. If no relationship exists, my Current Employer is not listed.

K. Plomaritas reports the following:
Employer: C.S. Mott Children's Hospital, Univeristy of Michigan Health System

I understand that the information above will be published within the journal article, if accepted, and that failure to comply and/or to accurately and completely report the potential financial conflicts of interest could lead to the following: 1) Prior to publication, article rejection, or 2) Post-publication, sanctions ranging from, but not limited to, issuing a correction, reporting the inaccurate information to the authors' institution, banning authors from submitting work to ASN journals for varying lengths of time, and/or retraction of the published work.

Name: Kathryn S. Plomaritas
Manuscript ID: K360-2024-000578R1
Manuscript Title: The Landscape of Pediatric Acute Care Nephrology Programs: A National Survey from the American Society of Pediatric Nephrology
Date of Completion: September 9, 2024
Disclosure Updated Date: August 29, 2024

ASN JASN Journal of the American Society of Nephrology KIDNEY360 Accessing Our World From Every Angle CJASN Clinical Journal of the American Society of Nephrology
American Society of Nephrology

ASN Journal Disclosure Form

As per ASN journal policy, I have disclosed any financial relationships or commitments I have held in the past 36 months as included below. I have listed my Current Employer below to indicate there is a relationship requiring disclosure. If no relationship exists, my Current Employer is not listed.

A. Riley reports the following:
Employer: Radgen Sports Management (husband)

I understand that the information above will be published within the journal article, if accepted, and that failure to comply and/or to accurately and completely report the potential financial conflicts of interest could lead to the following: 1) Prior to publication, article rejection, or 2) Post-publication, sanctions ranging from, but not limited to, issuing a correction, reporting the inaccurate information to the authors' institution, banning authors from submitting work to ASN journals for varying lengths of time, and/or retraction of the published work.

Name: Alyssa A. Riley
Manuscript ID: K360-2024-000578R1
Manuscript Title: The Landscape of Pediatric Acute Care Nephrology Programs: A National Survey from the American Society of Pediatric Nephrology
Date of Completion: August 28, 2024
Disclosure Updated Date: August 28, 2024

ASN American Society of Nephrology
JASN Journal of the American Society of Nephrology
KIDNEY360 Accessing Our World From Every Angle
CJASN Clinical Journal of the American Society of Nephrology

ASN Journal Disclosure Form

As per ASN journal policy, I have disclosed any financial relationships or commitments I have held in the past 36 months as included below. I have listed my Current Employer below to indicate there is a relationship requiring disclosure. If no relationship exists, my Current Employer is not listed.

D. Selewski reports the following:
Employer: Medical University of South Carolina; Consultancy: Pharmacosmos; and Research Funding: Travere Therapeutics, Inc.

I understand that the information above will be published within the journal article, if accepted, and that failure to comply and/or to accurately and completely report the potential financial conflicts of interest could lead to the following: 1) Prior to publication, article rejection, or 2) Post-publication, sanctions ranging from, but not limited to, issuing a correction, reporting the inaccurate information to the authors' institution, banning authors from submitting work to ASN journals for varying lengths of time, and/or retraction of the published work.

Name: David T. Selewski
Manuscript ID: K360-2024-000578R1
Manuscript Title: The Landscape of Pediatric Acute Care Nephrology Programs: A National Survey from the American Society of Pediatric Nephrology
Date of Completion: August 28, 2024
Disclosure Updated Date: March 28, 2024

ASN American Society of Nephrology | JASN Journal of the American Society of Nephrology | KIDNEY360 Accessing Our World From Every Angle | CJASN Clinical Journal of the American Society of Nephrology

ASN Journal Disclosure Form

As per ASN journal policy, I have disclosed any financial relationships or commitments I have held in the past 36 months as included below. I have listed my Current Employer below to indicate there is a relationship requiring disclosure. If no relationship exists, my Current Employer is not listed.

V. Shih reports the following:
Employer: Childrens Hospital Colorado

I understand that the information above will be published within the journal article, if accepted, and that failure to comply and/or to accurately and completely report the potential financial conflicts of interest could lead to the following: 1) Prior to publication, article rejection, or 2) Post-publication, sanctions ranging from, but not limited to, issuing a correction, reporting the inaccurate information to the authors' institution, banning authors from submitting work to ASN journals for varying lengths of time, and/or retraction of the published work.

Name: Vivian Shih
Manuscript ID: K360-2024-000578R1
Manuscript Title: The Landscape of Pediatric Acute Care Nephrology Programs: A National Survey from the American Society of Pediatric Nephrology
Date of Completion: September 6, 2024
Disclosure Updated Date: September 6, 2024

ASN
American Society of Nephrology
JASN
Journal of the American Society of Nephrology
KIDNEY360
Accessing Our World From Every Angle
CJASN
Clinical Journal of the American Society of Nephrology

ASN Journal Disclosure Form

As per ASN journal policy, I have disclosed any financial relationships or commitments I have held in the past 36 months as included below. I have listed my Current Employer below to indicate there is a relationship requiring disclosure. If no relationship exists, my Current Employer is not listed.

K. Short reports the following:
Employer: Children's of Alabama; and Patents or Royalties: Intellectual property with UAB for CRRT machine set ups and modifications.

I understand that the information above will be published within the journal article, if accepted, and that failure to comply and/or to accurately and completely report the potential financial conflicts of interest could lead to the following: 1) Prior to publication, article rejection, or 2) Post-publication, sanctions ranging from, but not limited to, issuing a correction, reporting the inaccurate information to the authors' institution, banning authors from submitting work to ASN journals for varying lengths of time, and/or retraction of the published work.

Name: Kara C Short
Manuscript ID: K360-2024-000578R1
Manuscript Title: The Landscape of Pediatric Acute Care Nephrology Programs: A National Survey from the American Society of Pediatric Nephrology
Date of Completion: September 9, 2024
Disclosure Updated Date: September 9, 2024
