# Supplementary material for: The Landscape of Pediatric Acute Care Nephrology Programs: A National Survey from the American Society of Pediatric Nephrology
Source: Kidney360. 2024 Sep 26;5(11):1713–7. doi: 10.34067/KID.0000000593 (PMC12282623; doi:10.34067/KID.0000000593)
Supplement: SUPPLEMENTARY MATERIAL [file kidney360-5-1713-s002.pdf]

Pediatric Acute Renal Replacement Survey

The following survey is aimed at assessing current practices in the provision of pediatric acute renal replacement therapies. We want to assess the types of therapies and the resources available across different ICU's. Importantly, we are looking to evaluate the service that are focused in the acute setting (not the chronic dialysis program).

Please include the name of your hospital as well as the name and e-mail of the person completing the survey to ensure only one response per hospital is submitted.

Name of your hospital: __________________________________

Name and title/position for person completing this survey: ____

Email address for the person completing this survey: ____

1 Does your hospital have an Acute Renal Replacement/Kidney Therapies Program?

(Ie: defined as a dedicated program with leadership/resources, directed at overseeing the delivery of acute RRT, and focused on critical care nephrology and/or ICU patients)

- ◯ Yes
- ◯ No

1a How is your acute kidney therapy/CRRT program administered at your hospital?

- ◯ Through Pediatric Nephrology only
- ◯ Through ICU only
- ◯ Jointly
- ◯ Other, please specify

If other, please specify: __________________________________

2 Does your hospital have a medical director overseeing acute kidney therapy?

- ◯ Yes
- ◯ No

2a Is the medical director a:

- ☐ Pediatric Nephrologist
- ☐ Critical Care Physician
- ☐ Other; please specify

If other, please specify: __________________________________

2b How much effort (% FTE) does the director at your hospital have allocated for this position?

0 50% 100%

(Place a mark on the scale above)

3 In your opinion, how much effort (% FTE) is required for an acute care nephrology director to provide appropriate oversight of the program?

0 50% 100%

(Place a mark on the scale above)

4 Does your hospital have an acute care dialysis nursing director?

◯ Yes
◯ No

4a How much effort does the nursing director have allocated for this position?

0 50% 100%

(Place a mark on the scale above)

5 In your opinion, how much effort (% FTE) is required for an acute care nephrology nursing director to provide appropriate oversight of the program?

0 50% 100%

(Place a mark on the scale above)

6 Do you have an APP dedicated to help provide acute RRT?

◯ Yes
◯ No

6a How many FTE(s) are dedicated to APPs in your acute care program?

____

7 Do you have any other leadership roles associated with providing acute renal replacement therapy at your hospital? Check all that apply:

☐ Nurse educator
☐ Program administrator
☐ Quality improvement leader
☐ Other; please specify

If other; please specify (including role/title and % FTE if applicable):

____

8 Where do you perform extracorporeal CRRT? Check all that apply:

☐ NICU
☐ PICU
☐ CICU
☐ Other; please specify

If other, please specify:

____

9 Where do you perform acute PD? Check all that apply:

☐ NICU
☐ PICU
☐ CICU
☐ Other; please specify

If other, please specify:

____

10 Is your acute care program administered jointly, or as a part of, your chronic dialysis program?

◯ Yes
◯ No

11 Is your acute care nursing team and your chronic nursing team joined in tasks/activities/call?

◯ Yes
◯ No

12 What modalities of acute RRT are performed at your hospital? Check all that apply:

- ☐ CRRT (or PIRRT) with Prismax,
- ☐ CRRT (or PIRRT) with Prismaflex
- ☐ CRRT (or PIRRT) with NxStage
- ☐ SCUF via in-line hemofilter with ECMO
- ☐ SCUF/Ultrafiltration with Aquadex
- ☐ CRRT (or PIRRT) with Aquadex for modified CVVH
- ☐ CRRT (or PIRRT) with Carpediem
- ☐ Acute Intermittent (or PIRRT) with HD
- ☐ Acute Peritoneal Dialysis - Manual
- ☐ Acute (and/or chronic) PD - Cycler
- ☐ Other; please specify

If other, please specify: ______________________

13 What other extracorporeal therapies are managed by Nephrology in your hospital? Check all that apply:

- ☐ Plasmapheresis
- ☐ Photopheresis
- ☐ Lipid/LDL Apheresis
- ☐ RBC Apheresis
- ☐ WBC Depletion
- ☐ SPAD (Single Pass Albumin Dialysis)
- ☐ MARS (Molecular Absorbent Recirculating System)
- ☐ ECMO (Extracorporeal Membrane Oxygenation)
- ☐ None of the above
- ☐ Other; please specify

If other, please specify: ______________________

14 What division/department is your Apheresis program part of? Check all that apply:

- ☐ Acute Care Program
- ☐ Chronic Dialysis Program
- ☐ Pediatric Nephrology
- ☐ Critical Care
- ☐ Blood Bank/Pathology
- ☐ Other; please specify
- ☐ We don't have an Apheresis program

If other, please specify: ______________________

15 Who is responsible for writing the CRRT prescriptions at your hospital?

- ☐ Pediatric Nephrology Provider
- ☐ Critical Care/ICU Provider
- ☐ Other; please specify

If other, please specify: ______________________

16 Who sets up CRRT machines in your hospital? Check all that apply:

- ☐ Dialysis nurses that also works in a chronic dialysis unit
- ☐ Dialysis nurses who just provide acute care therapies
- ☐ ICU bedside nurses
- ☐ Resource ICU nurses (ie: specially trained ICU RN)
- ☐ Resource nurses (ie: specially trained RN who has other duties)
- ☐ ECMO specialists
- ☐ Other; please specify

If other, please specify: ______________________

17	Do the nurses who set up the CRRT machines train in chronic hemodialysis before being allowed to do CRRT therapies in your hospital?	◯ Yes<br>◯ No
18	Do you have nurses solely dedicated to the acute care program?	◯ Yes<br>◯ No
18a	How many nursing FTE(s) do you have dedicated solely to your acute care program?	________________________
19	Over the last 3 years, what is the estimated/average number of patient days of CRRT per year at your hospital?	◯ < 250<br>◯ 250 - 499<br>◯ 500 - 745<br>◯ 750 - 999<br>◯ > 1000<br>◯ Unknown
20	Do you perform acute PD in neonates < 30 days of age?	◯ Yes<br>◯ No
21	Do you perform CRRT in neonates < 30 days of age?	◯ Yes<br>◯ No
21a	Which CRRT modalities are available at your hospital for neonates < 30 days of age? Check all that apply:	☐ Aquadex with ultrafiltration only<br>☐ Aquadex with modified CVVH<br>☐ Carpediem<br>☐ Prismax/Prismaflex<br>☐ Acute PD<br>☐ Other; please specify
	If other, please specify:	________________________
22	As it relates to care of neonates (< 30 days), which best describes your program?	◯ No plan to start a neonatal CRRT program<br>◯ Plan to start a neonatal CRRT program in the next 12 months<br>◯ In the process of starting a neonatal CRRT program<br>◯ Established a program within the last 12 months<br>◯ Have an established program in place for > 1 year<br>◯ Other; please specify
	If other, please specify:	________________________
23	Who provides specialized nursing coverage (ie: trouble-shooting, machine support, etc) for patients in your hospital on CRRT? Check all that apply:	☐ Dialysis trained nurses who do acute/chronic<br>☐ Dialysis trained nurses who do acute only<br>☐ Resource (specialized) nurses<br>☐ ECMO Specialists<br>☐ Other; please specify
	If other, please specify:	________________________
24	Do you have a QI Program dedicated to patients receiving acute RRT?	◯ Yes<br>◯ No
24a	Do you track any data for patients receiving acute RRT?	◯ Yes<br>◯ No

24b What patient related metrics do you track? Check all that apply:

- ☐ Patients by year
- ☐ Patients by location/unit
- ☐ Patient age
- ☐ Patient weight
- ☐ Patient diagnosis
- ☐ Reason for requiring acute RRT
- ☐ Percent fluid overload at RRT initiation
- ☐ Patient survival
- ☐ Other; please specify

If other, please specify:

____

24c What circuit related metrics do you track? Check all that apply:

- ☐ Type of machine
- ☐ Type of filter
- ☐ Type of prime
- ☐ Modality - CRRT vs PIRRT vs PD vs iHD
- ☐ Circuit life
- ☐ Effluent rate
- ☐ Fluid removal goals
- ☐ Instability during initiation
- ☐ Unanticipated down time
- ☐ Anticoagulation
- ☐ Other; please specify

If other, please specify:

____

24d What access related metrics do you track? Check all that apply:

- ☐ Catheter size
- ☐ Catheter location
- ☐ Cuffed vs uncuffed
- ☐ Complications
- ☐ Other; please specify

If other, please specify:

____

24e What do you see as major barriers to implementing a QI program dedicated to acute RRT? Check all that apply:

- ☐ Lack of interest from providers
- ☐ Lack of resources/infrastructure (ie: data analytics, etc)
- ☐ Lack of staff/dedicated time
- ☐ Lack of support from leadership
- ☐ Lack of expertise/training
- ☐ Lack of standardized practices
- ☐ Low patient volume
- ☐ Other; please specify

If other, please specify:

____

25 Do you have a standardized education program for bedside nurses prior to first use of CRRT?

- ◯ Yes
- ◯ No

26 What does your initial education/training program for bedside nurses include? Check all that apply:

☐ Bedside orientation
☐ Didactics
☐ Hands-on skill practice
☐ High fidelity simulation
☐ Other; please specify
☐ Specific training is not provided

If other, please specify:

____

27 Do you have a standardized education program for maintaining CRRT competencies for bedside nurses?

◯ Yes
◯ No

28 What does your education/training to maintain CRRT competencies for bedside nurses include? Check all that apply:

☐ Bedside orientation
☐ Didactics
☐ Hands-on skill practice
☐ High fidelity simulation
☐ Other; please specify
☐ Specific training is not provided

If other, please specify:

____

29 What frequency is education/training for maintaining CRRT competencies offered for bedside nurses?

◯ Yearly, or more frequently
◯ Every two years
◯ Asynchronous (ie: as needed intervals)
◯ Not offered
◯ Other; please specify

If other, please specify:

____

30 Do you have minimum criteria to evaluate CRRT competencies for nurses?

◯ Yes
◯ No

31 Do you have minimum criteria to evaluate CRRT competencies for providers?

◯ Yes
◯ No

32 Do you have a dedicated pharmacist supporting CRRT at your hospital (ie: with training/experience specific to CRRT)?

◯ Yes
◯ No

32a Do you have any pharmacy support (ie: ICU pharmacist) for patients receiving CRRT?

◯ Yes
◯ No

33 Do you have a dietician with training/experience specific to CRRT?

◯ Yes
◯ No

34 Does your program have a vascular access protocol for catheter selection and placement?

◯ Yes
◯ No

35 Who places vascular access for acute dialysis in your hospital? Check all that apply:

☐ Critical Care
☐ Interventional Radiology
☐ Pediatric Surgery
☐ Other; please specify

If other, please specify: __________________________________

36 When do you consider a cuffed line for acute care therapy (assuming no other contraindications)?

- ☐ Immediately in patients likely to be on longer than 1 week
- ☐ Immediately in patients likely to be on longer than 2 weeks
- ☐ After 1 week of RRT
- ☐ After 2 weeks of RRT
- ☐ When the patient is transitioned to intermittent HD
- ☐ Other; please specify

If other, please specify: ____

37 What types of anticoagulation are routinely used at your hospital for extracorporeal CRRT? Check all that apply:

- ☐ Regional Citrate/Calcium Anticoagulation
- ☐ Systemic Heparin (Standard Unfractionated Heparin)
- ☐ Regional Heparin/Protamine Anticoagulation
- ☐ Prostacyclin
- ☐ Direct Thrombin Inhibitors (ie: Bivalirudin, Hirudin, Argatroban, etc)
- ☐ Serine Protease Inhibitors (ie: Nafamostat)
- ☐ Low Molecular Weight Heparin
- ☐ Other; please specify

If other, please specify: __________________________________

38 If there is any other information or feedback you would like to provide, please let us know here! ____
